# Training of Working Memory Impacts Neural Processing of Vocal Pitch Regulation

**DOI:** 10.1038/srep16562

**Published:** 2015-11-10

**Authors:** Weifeng Li, Zhiqiang Guo, Jeffery A. Jones, Xiyan Huang, Xi Chen, Peng Liu, Shaozhen Chen, Hanjun Liu

**Affiliations:** 1 Department of Rehabilitation Medicine, The First Affiliated Hospital, Sun Yat-sen University, Guangzhou, 510080, P. R. China; 2 Department of Biomedical Engineering, School of Engineering, Sun Yat-sen University, Guangzhou, China, 510006; 3 Psychology Department and Laurier Centre for Cognitive Neuroscience, Wilfrid Laurier University, Waterloo, Ontario, N2L 3C5, Canada

## Abstract

Working memory training can improve the performance of tasks that were not trained. Whether auditory-motor integration for voice control can benefit from working memory training, however, remains unclear. The present event-related potential (ERP) study examined the impact of working memory training on the auditory-motor processing of vocal pitch. Trained participants underwent adaptive working memory training using a digit span backwards paradigm, while control participants did not receive any training. Before and after training, both trained and control participants were exposed to frequency-altered auditory feedback while producing vocalizations. After training, trained participants exhibited significantly decreased N1 amplitudes and increased P2 amplitudes in response to pitch errors in voice auditory feedback. In addition, there was a significant positive correlation between the degree of improvement in working memory capacity and the post-pre difference in P2 amplitudes. Training-related changes in the vocal compensation, however, were not observed. There was no systematic change in either vocal or cortical responses for control participants. These findings provide evidence that working memory training impacts the cortical processing of feedback errors in vocal pitch regulation. This enhanced cortical processing may be the result of increased neural efficiency in the detection of pitch errors between the intended and actual feedback.

Working memory refers to the neural process by which information is stored and manipulated over a brief period of time^1^. Multiple lines of evidence have demonstrated strong associations between working memory and complex cognitive tasks such as fluid reasoning^2^,^3^, reading comprehension^4^,^5^, and attentional control^6^,^7^. Brain damage due to events such as stroke and traumatic brain injury, as well as developmental and psychiatric disorders such as intellectual development disorder and schizophrenia, can cause impairments in working memory that affect quality of life^8^,^9^. Given the importance of working memory in facilitating complex cognition, recent years have seen a surge in the development of training programs aimed at not only enhancing working memory capacity, but also producing effects that generalize to untrained tasks.

Previous research has shown that working memory capacity can be improved by training. For example, following computerized working memory training, children with attention-deficit/hyperactivity disorder and patients who have recently suffered a stroke showed a significant improvement in their working memory capacity^10^,^11^. Moreover, working memory training can lead to beneficial effects in tasks that were not trained. For example, in a study by Jaeggi *et al*.^2^, participants were trained on a dual *n*-back task where they simultaneously heard a series of single letters while they saw a square sequentially placed at different positions on a screen. Their task was to determine whether each of these stimuli was presented n items back in the series. After this training, participants exhibited improved performance on an untrained task that involved non-verbal reasoning. Similarly, Dahlin *et al*.^12^ reported that, after participating in five weeks of adaptive training that required participants to update single items (letters, digits, colors, and spatial locations) in their working memory, young adults significantly improved their performance on a non-trained 3-back working memory task.

In addition to its influence on the above-mentioned cognitive functions, working memory has been found to be involved in sensorimotor integration in speech processing. In the phonological loop system of Baddeley and Hitch’s working memory model^1^, verbal information is processed through the interaction between the passive phonological storage and the active articulatory rehearsal. It has been suggested that sensorimotor processes may assist with the representation and manipulation of information, and auditory working memory acts to translate the auditory information into a rehearseable sensorimotor code^13^. Previous research has established a neural network underlying auditory verbal working memory, which includes the dorsolateral prefrontal cortex, premotor cortex, superior temporal gyrus, and area Spt (Sylvian-parietal-temporal)^14^,^15^; activity in these structures was also observed for the auditory-motor processing of feedback errors during vocal pitch regulation^16^,^17^,^18^. In particular, area Spt has been suggested to act as an auditory-motor interface for working memory because of its involvement in the temporary storage of verbal information during working memory tasks^19^, and the mapping of perceived speech sounds onto articulatory representations^20^. It is thus proposed that the internal representation and manipulation of speech sounds stored in working memory rely on auditory-motor integration^13^.

There are theories that postulate that auditory-motor integration in speech processing relies on a subtractive comparison of the expected auditory feedback (corollary discharge), based on a copy of the motor command, with the actual vocal output (re-afference)^21^,^22^. In the case of vocal pitch regulation, errors heard in auditory feedback may be detected by comparing the auditory re-afference with a sensory memory trace of the vocal target, with perceived discrepancies eliciting a corrective motor command to regulate the vocal production^23^. Working memory functions to integrate incoming information with stored information in existing memory^24^, and it has been suggested that information related to the speech motor command and sensory re-afference can be stored in short-term memory within a feedback circuit and recalled when needed to adjust the motor activity^25^,^26^. On the other hand, activation of the brain’s error detection system is affected by characteristics of the working memory system^27^, and the error detection mechanisms can benefit from training of working memory, as evidenced by increased amplitudes of the error-related negativity (ERN) using electroencephalography (EEG) for participants who received a training of auditory, visual, and cross-modality working memory skills using the CogniFit Personal Coach Program^28^. Moreover, there is evidence that intensive training of auditory working memory can make the storage, access, updating, and rehearsal of auditory information more efficient^29^. Thus, training of working memory may increase neural efficiency in the detection of feedback errors during the online monitoring of self-produced vocalization, facilitating the cortical mechanisms of auditory-motor integration for voice control.

There is also evidence that training individuals to recognize speech sounds involves a variety of cognitive abilities such as working memory and acoustic discrimination, and that this perceptual training can drive changes to speech motor control. For example, in one recent speech perceptual learning study, Mandarin speakers were trained to associate pictures with words that were spoken with pitch patterns that resembled five Thai tones. Participants who received the training produced significantly smaller N1 responses and larger P2 responses to pitch errors in their voice auditory feedback, while control participants who did not undergo the training did not^30^. Given that improving perceptual abilities can directly impact working memory^31^, it is also possible that changes in sensorimotor integration in speech processing caused by perceptual training are related to improvements in working memory capabilities. To date, no study has directly investigated the interactions between working memory training and plasticity in auditory-motor integration.

Since the training of auditory working memory can lead to increased neural efficiency in the storage and manipulation of auditory information, it is conceivable that auditory working memory training may also lead to beneficial effects for voice control by facilitating the detection of vocal pitch errors in auditory feedback. To answer this question, the present event-related potential (ERP) study recruited one trained group who participated in an adaptive training of auditory working memory using a digit span backwards (DSB) paradigm, and one control group who did not receive any training. At the pre-training and post-training sessions, both trained and control participants were instructed to sustain a vowel phonation while hearing their voice auditory feedback unexpectedly pitch-shifted. Behavioral and cortical responses to pitch perturbations were measured to examine the influence of working memory training on the auditory-motor control of vocal production. Because previous research has shown that working memory training leads to beneficial effects on untrained tasks^2^,^12^, and changes in N1 and P2 responses to vocal pitch perturbations following speech perceptual learning^30^, we predicted that this adaptive DSB training would result in not only improvements in working memory capacity, but also improved auditory-motor processing of vocal pitch. Specifically, we expected larger vocal responses, smaller N1 responses (i.e., less negative), and larger P2 responses (i.e., more positive) to pitch feedback perturbations for the participants who underwent the training.

## Methods

### Subjects

Thirty-three right-handed and native-Mandarin speaking participants, who were students at Sun Yat-sen University in China, were recruited and randomly assigned to the trained or control group. Eighteen participants (8 male and 10 female; mean age = 22 years) were assigned to the trained group and underwent 10 days of working memory training. Fifteen participants (7 male and 8 female; mean age = 21 years) who did not undergo the training were assigned to the control group. The two groups did not differ in terms of age, gender, and education. Participants had no history of language, hearing, or neurological disorders. Participants were included in the experiments only if they had normal hearing (≤25 dB hearing level [HL] pure-tone thresholds from 250–4000 Hz). Written informed consent was obtained from all participants, and the research protocol was approved by the Institutional Review Board of The First Affiliated Hospital at Sun Yat-sen University of China in accordance with the Code of Ethics of the World Medical Association (Declaration of Helsinki). The study was carried out in accordance with the approved guidelines.

### Working memory training

The training scheme was designed to provide participants with practice in auditory working memory. Working memory training was carried out in a sound-attenuated booth. Training was implemented with E-Prime software (Psychological Testing Services, Pittsburgh, Pennsylvania) using a DSB paradigm, during which participants heard a string of digits and then were asked to type the digits in the reverse order that they were presented (e.g., if the participant heard “1 2 3 4”, then the correct response was “4 3 2 1”). Participants were seated in front of a personal computer and heard the sounds through Sennheiser headphones (HMD 250). Listeners were allowed to choose a preferred loudness level for training stimuli at the outset of the experiment, and this level was maintained throughout training.

The duration of the experiment was 12 days for each participant. Both trained and control participants completed the pre- and post-test on the first and twelfth day, and the trained participants received training on the second through eleventh day (i.e. training days 1–10). The training paradigm was adaptive such that the length of digit span was adjusted based on the listener’s performance. On training day 1, the training session started with 2 digits. Each block consisted of 7 lists of digits, and 10 blocks were completed during each training day. Subjects had to reach 70% accuracy on a given span before the length of the spans was increased. Accuracy of lower than 50% caused the span lengths to decrease on the subsequent trial. Participants began each new training day with the span length they had attained on the last trial of the previous day.

Participants were asked to remember the digits one through nine, which were spoken by a native Mandarin speaker and recorded with a sampling frequency of 44100 Hz. The digit stimuli were then RMS matched to a 70 dB sound pressure level (SPL) pure tone at 1 kHz. Pilot testing revealed that listeners plateaued in performance when all training was presented in quiet, but not when noise was introduced. We therefore presented the digits in noise with varying signal-to-noise (SNR) levels. Steady-state noise matching the long-term average spectrum of the recordings was created using MATLAB and mixed with the target recordings in Adobe Audition to create stimuli with SNRs of −5 and −10 dB. In addition, a set of 27 non-speech sounds were obtained from online databases, including twelve animal sounds (e.g., bird song, dog bark), two mechanical sounds (e.g., clock ticking, gun shot), five non-speech human sounds (e.g., cough, laugh), and eight musical sounds (e.g., violin, flute). These sounds were cropped to 1 s, RMS matched in amplitude to the calibration tone, and then mixed with all target digits at −5 and −10 dB SNR, resulting in a total of 468 stimulus combinations.

**Figure 1:**
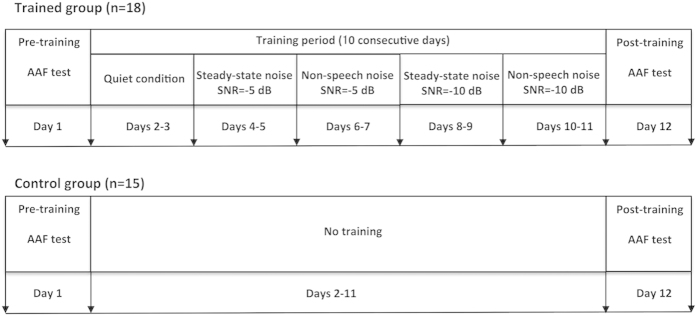
Schematic illustration showing the experimental setup. Trained participants received adaptive training using a digit span backwards paradigm over 10 consecutive days. The digits were presented in quiet, steady-state noise (−5 and −10 dB), and non-speech noise (−5 and −10 dB) conditions. Control participants did not receive training. Both groups participated in the altered auditory feedback (AAF) experiment before and after training, during which they produced vowel sounds while hearing their voice pitch feedback unexpectedly shifted through headphones.

The training paradigm was implemented over 10 consecutive days (see Fig. 1). On training days one and two, the digits were presented in a quiet condition (i.e. without noise). On training days three and four, each digit was presented in steady-state noise at −5 dB SNR. On training days five and six, each digit was presented in non-speech distractors that were randomly chosen and presented at −5 dB SNR. Training days seven through ten replicated training days three through six but at −10 dB SNR.

### Behavioral and neurophysiological assessment

The vocal production experiment was carried out using the altered auditory feedback (AAF) paradigm. Both trained and control participants were instructed to produce a steady vowel sound /u/for about 5–6 s at their habitual and comfortable pitch and loudness level, during which their voice pitch feedback was unexpectedly shifted +50 or +200 cents (100 cents = one semitone) for 200 ms, and fed back to them instantaneously. The first pitch-shifted stimulus was presented with a delay of 500–1000 ms after vocal onset, and the succeeding stimuli occurred with an inter-stimulus interval of 700–900 ms. There were five pitch-shift stimuli per vocalization and participants produced 40 consecutive vocalizations. In total, 200 trials were collected including 100 trials for +50 cents and 100 trials for +200 cents.

The vocal production experiment was conducted in a sound-attenuated booth. Prior to the testing, the experimental system was calibrated to ensure that the intensity of voice feedback heard by the participants was 10 dB SPL higher than that of the subjects’ voice output to partially mask any air-born and bone-conducted feedback^32^. The voice signals were transduced by a dynamic microphone (DM2200, Takstar Inc.) and amplified by a MOTU Ultralite Mk3 Firewire audio interface. The amplified signals were pitch-shifted through an Eventide Eclipse Harmonizer that was controlled by a custom-developed MIDI software program (Max/MSP, v.5.0 by Cycling 74) running on a Macintosh computer. This program was also used to manipulate parameters including the magnitude, direction, and duration of the pitch shifts and generate transistor-transistor logic (TTL) control pulses to signal the onset and offset of the pitch shifts. Finally, the pitch-shifted voices were amplified by an ICON NeoAmp headphone amplifier and fed back to the participants through insert earphones (ER1-14 A, Etymotic Research Inc.). The voice, feedback, and TTL control pulses were digitized at 10 kHz by a PowerLab A/D converter (ML880, AD Instruments), recorded using LabChart software (v.7.0 by AD Instruments), and saved onto another Macintosh computer.

EEG signals were recorded using a 64-electrode Geodesic Sensor Net, amplified by a Net Amps 300 amplifier (Electrical Geodesics Inc.), digitized at a sampling frequency of 1 kHz, and saved onto a Mac Pro computer. A DIN synch cable was used to send the TTL control pulses generated by Max/MSP to the EEG recording system for the measurement of stimulus-evoked potentials. During the online recording, EEG signals across all channels were referenced to the vertex (Cz). Impedance of individual sensors were adjusted and maintained below 50 kΩ^33^.

### Data analyses

#### Vocal Responses

The measurement of vocal responses to pitch-shifted voice auditory feedback was implemented using a custom-developed IGOR PRO program (v.6.0, Wavemetrics Inc.) using event-related averaging techniques^34^. Voice F_0_ contours in Hertz were extracted from voice signals using Praat^35^, and converted to the cent scale using the formula: cents =  100 × (12 × log_2_(F_0_/reference)) [reference = 195.997 Hz (G4)]. Voice F_0_ contours were then segmented into epochs from 200 ms before and 700 ms after the stimulus onset. All individual trials were visually inspected such that those trials containing vocal interruption or signal processing errors were excluded from analyses. Finally, artifact-free trials for each condition were averaged to generate an overall response. Acceptable responses exceeded a value of two standard deviations (SDs) of the pre-stimulus mean beginning at least 60 ms after the onset of the pitch shift and lasting at least 50 ms. The response magnitude was measured by subtracting the peak value of voice contour following the response onset from the pre-stimulus mean (−200 to 0 ms). The response latency was determined as the time of voice F_0_ departure from the pre-stimulus mean by more than 2 SDs.

#### Evoked Potentials

The EEG signals were analyzed off-line using NetStation software (v.4.5, Electrical Geodesics Inc.). All channels were digitally band-passed filtered with cut-off frequencies of 1 to 20 Hz. Individual trials were segmented into epochs with a window of −200 ms and +500 ms relative to the onset of the pitch shift. Segmented trials contaminated by excessive muscular activity, eye blinks, or eye movements were assessed using the Artifact Detection Toolbox in NetStation and excluded from further analyses. An additional visual inspection was also performed on individual trials to ensure that artifacts were being adequately rejected. Individual electrodes were excluded from further analyses if they contained artifacts more than 20% of the segmented trials. Finally, artifact-free segments were averaged, re-referenced to the average of the electrodes on each mastoid, and baseline-corrected across all conditions. Given that cortical responses to pitch-shifted voice auditory feedback are mostly pronounced in the N1-P2 complex^36^,^37^, the amplitudes and latencies of N1 and P2 were measured as the negative and positive peaks in the time windows of 80–180 ms and 160–280 ms after the onset of the pitch shift and submitted to statistical analyses.

The DSB scores and the magnitudes and latencies of vocal and neurophysiological responses were subjected to repeated-measures mixed analyses of variance (ANOVAs) using SPSS (v.16.0). Testing session (pre- vs. post-training), stimulus magnitude (+50 and +200 cents), and electrode site (FC1, FC3, FCz, FC2, FC4, C1, C3, Cz, C2, C4, P1, P3, Pz, P2, P4) were chosen as within-subject factors, while group (trained vs. control group) was chosen as a between-subject factor. Appropriate subsidiary RM-ANOVAs were calculated if higher-order interactions reached significance. Greenhouse-Geisser was used to correct probability values for multiple degrees of freedom when violations of the sphericity assumption occurred.

## Results

### DSB measure

**Figure 2:**
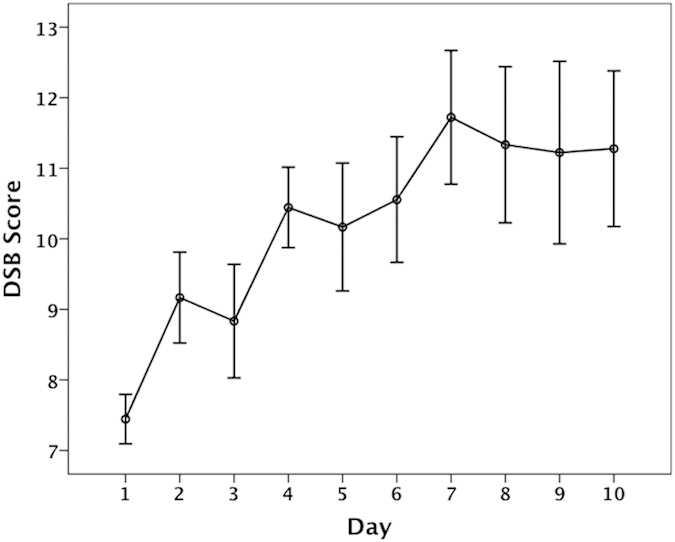
The mean scores of the digit span backwards task across the 10 training days. The error bars represent the standard deviations of the mean scores.

Fig. 2 shows participants’ performance indexed by the DSB scores throughout training. As can be seen, the DSB score was below 10 (ranging from 7.4 to 9.2) on training days one through three. On training days four through ten, the DSB scores increased to as large as 11.7. A one-way ANOVA revealed a significant main effect of testing day (F(9, 153) = 22.288, p < 0.001), and post-hoc Bonferroni comparisons showed that the DSB scores on training days one through three were significantly lower than those on training days seven through ten (p < 0.03). In summary, the significant increase in the DSB measure indicates improvements in working memory capacity following training.

### Vocal response

One two-way mixed ANOVA conducted on the vocal response magnitude revealed no significant main effects of testing session (F(1, 31) = 0.045, p = 0.834) (pre-training: 14.7 ± 6.3 cents; post-training: 13.6 ± 5.8 cents), stimulus magnitude (F(1, 31) = 0.963, p = 0.334) (+50 cents: 14.0 ± 5.6 cents; +200 cents: 14.2 ± 6.4 cents), or group (F(1, 31) = 1.055, p = 0.312) (trained: 13.6 ± 5.6 cents; control: 14.7 ± 6.5 cents). Similarly, response latencies did not differ as a function of testing session (F(1, 31) = 0.425, p = 0.519) (pre-training: 77 ± 24 ms; post-training: 75 ± 18 cents), stimulus magnitude (F(1, 31) = 0.234, p = 0.632) (+50 cents: 75 ± 19 ms; +200 cents: 77 ± 23 ms), or group (F(1, 31) = 0.054, p = 0.817) (trained: 76 ± 21 ms; control: 77 ± 21 ms). In addition, no significant interactions between these factors were observed for response magnitude or latency (p > 0.05).

### ERP findings

**Figure 3:**
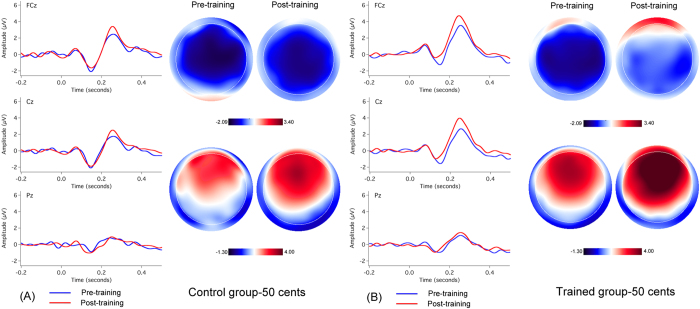
Grand-averaged ERP waveforms (FCz, Cz, Pz) and topographical distributions of N1 and P2 amplitudes in response to pitch shifts of +50 cents across all control (**A**) and trained participants (**B**) before (blue solid lines) and after (red solid lines) training.

**Figure 4:**
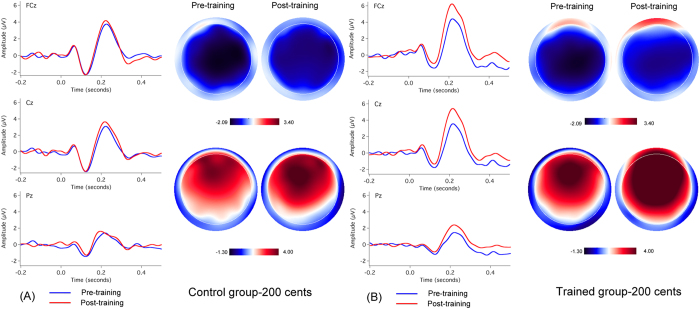
Grand-averaged ERP waveforms (FCz, Cz, Pz) and topographical distributions of N1 and P2 amplitudes in response to pitch shifts of +200 cents across all control (**A**) and trained participants (**B**) before (blue solid lines) and after (red solid lines) training.

Fig. 3 and 4 show the grand-averaged ERP waveforms and topographical distributions of N1 and P2 amplitudes in response to pitch shifts of +50 and +200 cents across all control and trained participants before (blue solid lines) and after (red solid lines) working memory training. As can be seen, the cortical responses to pitch shifts produced by the control participants during the pre-training session did not differ from the responses produced during the post-training session. By contrast, following training N1 amplitudes decreased and P2 amplitudes increased in response to both the +50 and +200 cents pitch shifts heard by the trained participants, and these changes were primarily observed in the frontocentral electrodes.

**Figure 5:**
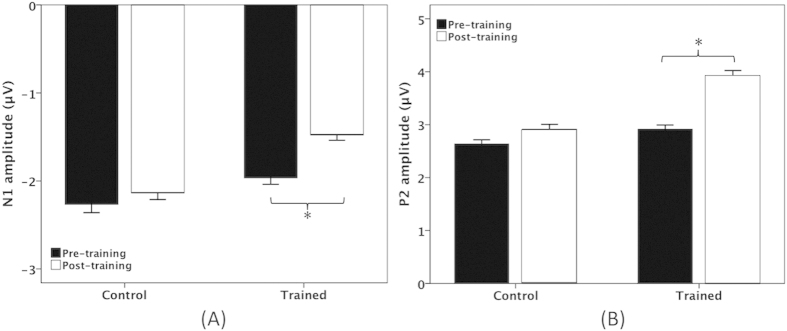
T-bar plots (means and standard errors) of N1 (**A**) and P2 (**B**) amplitude as a function of testing session and group. The black and the blank bars denote the cortical responses at the pre-training and the post-training session, respectively. The asterisks indicate significant differences between conditions.

One four-way mixed ANOVA conducted on the N1 amplitudes revealed significant main effects of testing session (F(1, 31) = 4.846, p = 0.035) and electrode site (F(14, 434) = 13.265, p < 0.001), whereas the main effects of stimulus magnitude (F(1, 31) = 3.209, p = 0.083) and group (F(1, 31) = 1.134, p = 0.295) failed to reach significance. There was also a significant testing session × electrode site × group interaction (F(14, 434) = 2.705, p = 0.038). Follow-up three-way mixed ANOVAs conducted on the data from the trained group revealed a significant decrease in N1 amplitudes (less negative) following training (F(1, 17) = 5.039, p = 0.038), whereas there was no systematic change in N1 amplitudes as a function of testing session for the control group (F(1, 14) = 0.650, p = 0.434) (see Fig. 5A).

As for N1 latency, pitch shifts of +50 cents elicited significantly longer N1 latencies than pitch shifts of +200 cents (143 ± 25 vs. 122 ± 20 ms) (F(1, 20) = 81.329, p < 0.001). There was also a significant main effect of electrode site (F(14, 434) = 3.785, p = 0.008), which was primarily caused by longer N1 latencies at electrode C4 as compared to Pz (p = 0.010) and P2 (p = 0.004). A main effect of group (F(1, 31) = 1.014, p = 0.322) and testing session (F(1, 31) = 1.486, p = 0.232), however, failed to reach significance. No significant interactive effects between these factors were found either (p > 0.05).

One four-way mixed ANOVA conducted on the P2 amplitudes revealed a significant main effect of stimulus magnitude (F(1, 31) = 20.102, p < 0.001), indicating that pitch shifts of +50 cents elicited significantly smaller P2 amplitudes than pitch shifts of +200 cents. There was also a significant main effect of electrode site (F(14, 434) = 73.727, p < 0.001), which was primarily driven by larger P2 amplitudes at frontocentral electrodes than parietal electrodes (p < 0.05) (see Figs 3 and 4). The main effect of testing session (F(1, 31) = 15.256, p < 0.001) reached significance, whereas there was no main effect of group (F(1, 31) = 2.928, p = 0.097). A significant interaction, however, was found between testing session and group (F(1, 31) = 4.917, p = 0.034). A follow-up three-way mixed ANOVA conducted on the data from the trained group revealed a significant main effect of testing session (F(1, 17) = 17.828, p = 0.001), indicating that P2 amplitudes significantly increased following training (see Fig. 5B). By contrast, P2 amplitudes did not differ as a function of testing session (F(1, 14) = 1.614, p = 0.225) for the control group.

Regarding P2 latency, there was a significant main effect of stimulus magnitude (F(1, 31) = 97.498, p < 0.001) as reflected by significantly shorter P2 latencies elicited by pitch shifts of +200 cents, as compared to pitch shifts of +50 cents (225 ± 24 vs. 252 ± 25 ms). However, the main effects of testing session (F(1, 31) = 3.774, p = 0.061), electrode site (F(14, 434) = 1.817, p = 0.148), and group (F(1, 31) = 0.037, p = 0.849) failed to reached significance. There also were no significant interactions between these factors (p > 0.05).

**Figure 6:**
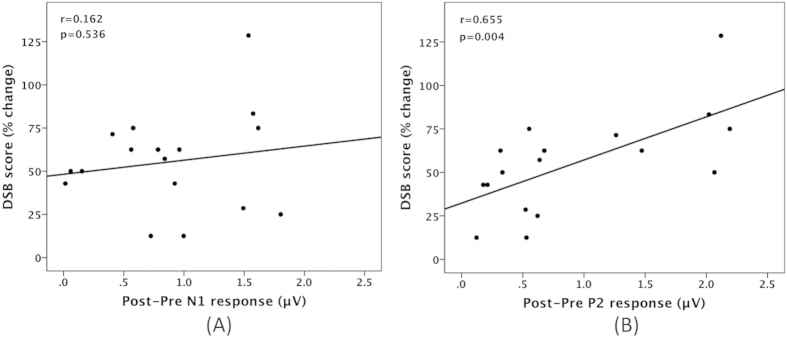
The percentage change of DSB scores is plotted against the post-pre difference for the mean N1 (**A**) and P2 (**B**) responses to pitch feedback perturbations. There was a significant positive correlation between the post-pre difference for the mean P2 responses and the percentage change in DSB scores (*r* = 0.655, p = 0.004), whereas the post-pre difference for the mean N1 responses was not significantly correlated with the percentage change in DSB scores (*r* = 0.162, p = 0.536).

In addition, regression analyses were performed to examine the relationship between working memory training and cortical responses to pitch-shifted voice auditory feedback. The percentage change of DSB scores (i.e. post-pre DSB scores divided by pre-training DSB scores) is plotted against the post-pre difference for the mean N1 and P2 responses in Fig. 6. The results showed a significant positive correlation between the post-pre difference for the mean P2 responses and the percentage change in DSB scores (*r* = 0.655, p = 0.004), indicating that the degree of improvement in working memory capacity was predictive of the training-related enhancement of cortical responses to pitch feedback perturbations. The post-pre difference for the mean N1 responses, however, was not significantly correlated with the percentage change in DSB scores (*r* = 0.162, p = 0.536).

## Discussion

The present study investigated whether working memory training can lead to improved processing of feedback errors during vocal pitch regulation. Before and after an adaptive DSB training procedure, participants’ vocal and cortical responses to errors detected in their auditory feedback were measured. As hypothesized, in addition to significant improvements in participants’ working memory capacity (i.e. the DSB score), we observed an impact of working memory training on the cortical processing of vocal pitch errors. The trained participants’ N1 responses to pitch errors in voice auditory feedback decreased, whereas the N1 responses for the first and second AAF sessions of control participants’ were not significantly different. Conversely, P2 responses increased after working memory training, but remained the same across the vocal production tasks for control participants. Moreover, the percentage change in DSB scores was significantly correlated with the post-pre difference for the mean P2 responses to pitch feedback perturbations, indicating that improvement in working memory capacity was predictive of training-related enhancement of cortical responses to voice feedback errors. Neither the trained participants nor the control participants, however, showed a systematic change in their vocal compensation magnitude or latency across the pre- and post-training sessions. Taken together, these findings demonstrate that training-related improvements in working memory capacities generalize to other tasks, and modify the cortical processing of mismatches between intended and actual auditory feedback.

### Training-related effects on the N1-P2 complex

A primary finding in this study is that the training of working memory led to decreased N1 responses when participants heard pitch-shifted auditory feedback. This finding is in line with other studies that showed decreased N1 amplitudes in response to pitch feedback perturbations after speech perceptual learning^30^, and decreased brain activity in the frontoparietal regions after working memory training^29^,^38^,^39^. The N1 component is generally thought to reflect pre-attentive detection of a mismatch between incoming auditory stimuli and the memory trace of previous sensory input into the auditory system^40^. We hypothesize that increased neural efficiency^41^ accounts for the decreased N1 responses to pitch errors in voice auditory feedback that we observed. This hypothesis is supported by work showing increased efficiency in the processing of auditory information following training of auditory working memory as reflected by decreases in brain activation. For example, in a recent study an adaptive *n*-back training with tonal sequences led to not only improved performance on an auditory 2-back task, but also decreased activation in the right inferior frontal gyrus and posterior parietal regions^29^. Thus, following intensive training of auditory working memory, fewer neural resources may be required for encoding the same level of auditory information such that the integration of incoming information with information stored in memory becomes more efficient. In the case of voice control then, the detection and comparison of pitch errors in voice auditory feedback may become more efficient, and this increased efficiency may be reflected by the significantly decreased N1 responses observed after training in the present study.

In addition to the changes in N1 responses, enhancement of P2 responses to pitch-shifted voice auditory feedback was also observed for trained participants. Similarly in a previous study, P2 responses to pitch feedback perturbations were significantly increased after a sound-to-word learning of lexical tones by associating speech stimuli with pictures of objects^30^. The present finding is also in line with other findings that showed increased theta power^42^ and ERN^28^ using EEG, and increased blood oxygenation level dependent (BOLD) responses measured with fMRI^43^,^44^,^45^ following working memory training. Since P2 responses to pitch feedback perturbations are associated with relatively later stages of cortical processing, it has been suggested that the P2 component may reflect functional mechanisms that demand higher-level cognitive processes of auditory-motor integration^46^. In particular, there is evidence that P2 receives contributions from the planum temporale^47^, and one function of this region is to support sensorimotor integration in speech processing^22^. For example, the posterior region of the planum temporale, area Spt, has been found to function as an interface between auditory and motor representations^20^,^48^. Taken together, increased P2 responses to pitch feedback errors may index the training-induced enhancement of the coordinated neural activity for the interaction between the auditory and motor systems in voice control.

Interestingly, the post-pre difference in the mean P2 responses was positively correlated with the percentage change in DSB scores, suggesting a relationship between the degree of improvement in working memory capacity and training-related enhancement of cortical responses to pitch feedback perturbations. This finding provides further evidence that working memory in the phonological loop is dependent on the operation of an auditory-motor interface system in the posterior planum temporale^19^. Also this correlation lends support to the idea that the P2 component reflects higher-level cognitive processing of feedback errors during the auditory-motor integration for voice control.

Since it has been previously shown that P2 responses become larger when participants attend to pitch feedback perturbations as compared to when they do not^49^,^50^, and that attentional control can be improved by working memory training^51^, one may argue that the training-related changes we observed in the N1-P2 complex were not the result of improved working memory capacities per se, but the result of an increased capacity for attentional control. That is, after working memory training, our trained participants may have paid more attention to their voice auditory feedback, and this increased attention caused the training-related changes in the N1-P2 complex. However, in addition to enhancing P2 responses, attending to pitch feedback perturbations has been shown to increase N1 amplitudes and increase the size of vocal compensations, as compared to when they are not attended^49^,^52^. In the present study, we observed *decreased* N1 responses and vocal compensation remained unchanged after training. Moreover, the correlation observed between the improvements in working memory capacity and the enhanced P2 responses to pitch perturbations suggests that the training gains in auditory-motor integration for voice control are most likely due to increased working memory capacity.

### The lack of training effect on the vocal compensation

Unlike the N1-P2 complex, the vocal compensations for the pitch errors heard in voice auditory feedback were not affected by the working memory training. Similarly, Chen *et al*.^30^ did not find a systematic change in the vocal responses to pitch feedback perturbations for participants who underwent training to associate unfamiliar speech sounds with words. These results are also in line with studies on visuomotor adaptation, which showed that improving working memory capacity using dual *n*-back training does not alter the rates of visuomotor adaptation^53^, although individual differences in spatial working memory capacity are predictive of the rate of early visuomotor adaptation^54^. In terms of the present study, there are a number of potential explanations for the lack of transfer to vocal compensation responses. For example, it is possible that working memory training may only benefit the detection of pitch errors in auditory feedback. It may also be that if participants were presented with feedback perturbations that were much smaller in magnitude than those presented in the present study, and near the threshold for detection, differences between a trained and untrained group of participants would emerge. In addition, there are several other factors that contribute to the underlying mechanisms engaged in the modulation of vocal compensation for voice feedback errors, such as stimulus features^55^,^56^ and task demands^57^,^58^. Future studies that manipulate these factors could provide a more thorough characterization of transfer effect of working memory training to auditory-motor integration.

### Limitation

One limitation of the present study was the use of a passive control group that was used to control for pre-/post-test effects, but did not receive any form of training between these tests. Thus, confounding factors such as motivational and psychological effects, which have been shown to influence the effectiveness of working memory training^59^, were not controlled using the present experimental design. Therefore, it is possible that transfer effects we observed are not solely a result of training, but may in part be related to different levels in motivation and/or practice. Positive transfer effects of working memory training will be more convincing when compared against an active control group with a cognitively engaging alternative intervention. Another limitation is the inclusion of a perception-in-noise component, which makes it difficult to determine how much of the transfer effect was a result of the working memory training gains or an improved ability to discriminate the acoustic stimuli in noise. In future studies, the present training paradigm should be compared to an adaptive DSB training without noise to determine the contributions of perception in noise vs. working memory capacity to the transfer effects we observed.

## Conclusions

Overall, we found neurophysiological evidence that training of auditory working memory impacts the auditory-motor processing of vocal pitch regulation at the cortical level. This includes decreased N1 and increased P2 responses to pitch errors in voice auditory feedback following training. Our results extend previous findings regarding the transfer of training gains in working memory, demonstrating that the cortical processing of vocal pitch regulation can benefit from working memory training.

## Additional Information

**How to cite this article**: Li, W. *et al*. Training of Working Memory Impacts Neural Processing of Vocal Pitch Regulation. *Sci. Rep.* **5**, 16562; doi: 10.1038/srep16562 (2015).
